# Return to Play After Thumb Ulnar Collateral Ligament Injuries Managed Surgically in Athletes—A Systematic Review

**DOI:** 10.1016/j.jhsg.2023.03.005

**Published:** 2023-03-31

**Authors:** Sachin Allahabadi, Jeffrey W. Kwong, Nirav K. Pandya, Steven S. Shin, Igor Immerman, Nicolas H. Lee

**Affiliations:** ∗ Department of Orthopaedic Surgery, University of California San Francisco, San Francisco, CA; † Department of Orthopaedic Surgery, Cedars-Sinai Medical Center, Los Angeles, CA

**Keywords:** Athlete, Return to play, Sport, Thumb, Ulnar collateral ligament

## Abstract

**Purpose:**

The purpose of this systematic review was to summarize the available data on how surgical management of injuries to the thumb ulnar collateral ligament (UCL) complex affects athletes and their return-to-play (RTP) and postinjury performance metrics in addition to evaluating rehabilitation guidelines.

**Methods:**

A systematic search was performed on PubMed and Embase databases for articles on outcomes of surgical treatment of thumb UCL injuries in athletes. Articles with expert recommendations on postoperative management and RTP guidelines were also included separately. Study characteristics were recorded, including sport, RTP rates, and data on performance. Recommendations were summarized by sport. The Methodological Index for Non-Randomized Studies (MINORS) criteria was used to assess methodological quality. The authors also present their recommended return-to-sport algorithm.

**Results:**

Twenty-three articles were included, including 11 with reports on patients and 12 expert opinions on guiding RTP. The mean MINORS score for the applicable studies was 9.4. In the 311 patients included, RTP was 98.1% in aggregate. No performance detriments were noted in athletes after surgery. Thirty-two (10.3%) patients had postoperative complications. The recommendations on timing to RTP vary by sport and author, but all recommended initial thumb protection when returning to sport. Newer techniques, such as suture tape augmentation, suggest the permission for earlier motion.

**Conclusions:**

Return-to-play rates after surgical treatment of thumb UCL injuries are high, with reassuring return to preinjury level of play with few complications. Recommendations for surgical technique have trended toward suture anchors and, now, suture tape augmentation with earlier motion protocols, although rehabilitation guidelines vary by sport and author. Current information on thumb UCL surgery in athletes is limited by the low quality of evidence and expert recommendations.

**Type of study/level of evidence:**

Prognostic IV.

Tears to the ulnar collateral ligament (UCL) of the thumb metacarpophalangeal (MCP) joint are commonly sustained sports injuries, with an incidence of approximately 50 per 100,000 emergency room visits per year.^1^ Occurring through extreme radial stress to the MCP joint, thumb UCL injuries most often result from avulsion of the ligament from its distal insertion on the proximal phalanx.^2^ These injuries can occasionally be complicated by avulsion fractures of the base of the proximal phalanx of the thumb and are sometimes associated with Stener lesions, in which the aponeurosis of the adductor pollicis becomes interposed between the UCL and its attachment site on the proximal phalanx.^3^^,^^4^

Stable injuries, or in other words grade I thumb UCL sprains (tenderness along the UCL without laxity) and grade II thumb UCL injuries (increased laxity with a firm end point on stress testing), may be successfully managed nonsurgically, and patients typically have no long-term pain or disability.^5^ Nonsurgical treatment typically involves immobilization, which may involve thumb spica casting (short-arm or hand-based), custom thermoplast splints, removable thumb spica splints, and functional braces.^6^ The period of immobilization before initiating motion exercises varies but is typically approximately 4 weeks, and the goal is to protect the MCP joint and reduce pain and inflammation.^5^, ^6^, ^7^ On the other hand, although partial thumb UCL tears can be treated by immobilization, Stener lesions and acute full thickness tears with instability are managed through surgical intervention because these UCL injuries frequently result in decreased pinch strength, instability, and reduced range of motion (ROM) of the thumb.^3^^,^^5^^,^^8^^,^^9^ These functional impairments and the goal of limiting long-term joint degeneration support surgical treatment for unstable injuries.

The importance of restoring stability and function is especially important in those who use their hands frequently. For example, in high-level athletes, this injury may affect performance and, therefore, job and career potential. Furthermore, if they require surgery, athletes may also lose playing time for wound and ligament healing in addition to immobilization after surgical management. Athletes also have increased risk for future injury during both noncontact and contact sports. Therefore, identifying an optimal surgical technique and rehabilitation protocol to facilitate return-to-play (RTP) in a safe and reliable manner is imperative, especially for athletes or those with substantial demands on their hands. Considerations for athletes include their hand dominance, specific sporting demands, practicality of playing with immobilization of the thumb, timing in season or career, and patient-specific goals.^7^ However, data on RTP after thumb UCL injuries in athletes have remained sparse and heterogeneous in the literature perhaps because of evolving surgical techniques, the unique demands of different sports and playing positions, and the difficulty of conducting comparative studies on elite athletes.

The purpose of this systematic review was to summarize the available data on how surgical management of injuries to the thumb UCL complex affects athletes and their RTP and postinjury performance metrics in addition to evaluating rehabilitation guidelines. The authors hypothesized that RTP would be high and athletes would have minimal performance detriments after surgery; furthermore, we hypothesized that the recommended timing for RTP would have substantial variation.

## Materials and Methods

**Figure 1:**
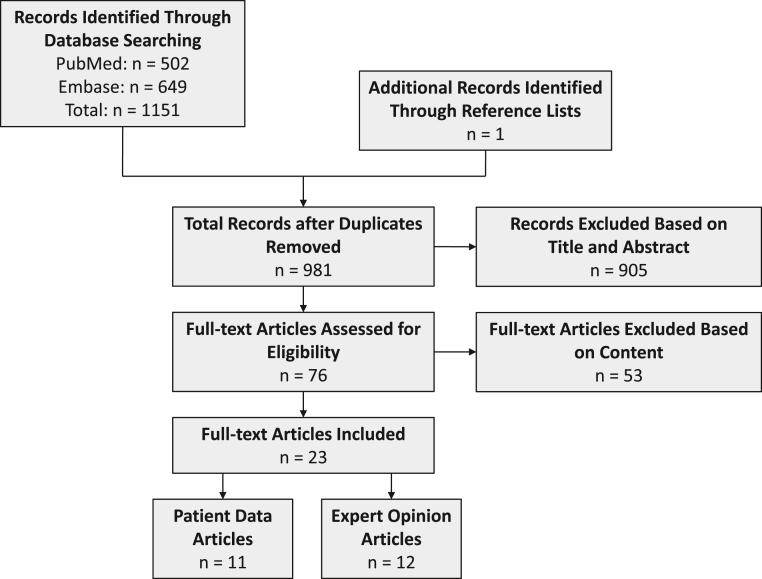
The Preferred Reporting Items for Systematic Reviews and Meta-Analyses (PRISMA) flowchart for study inclusion. Twenty-three full-text articles were included.

This systematic review was registered on the International Prospective Register of Systematic Reviews (CRD42022300157). The study adhered to Preferred Reporting Items for Systematic Reviews and Meta-Analyses (PRISMA) guidelines (Fig. 1). A systematic search using the PubMed and Embase databases was performed in December 2021 for the following search terms: (thumb) AND ((UCL) OR (ulnar collateral)). Studies were included if they were written in the English language and discussed surgical management of thumb UCL injuries in an athlete population, whether professional or nonprofessional. The authors sought data on RTP of sports and not return to work specifically. Expert opinions on management of athletes were included and summarized separately; the purpose of including these articles was to evaluate the variability in recommendations by technique. Review articles or technique articles without patients or expert opinion on management were excluded. Studies reporting on one patient (ie, case reports) were excluded from data analysis.

The screening and selection process was performed independently by two authors for inclusion (J.W.K. and S.A.) in a staged process from titles to abstracts to full-text review. Any article identified as eligible by one author was included in subsequent review.

Data collected from the included articles included publication characteristics, such as the year it was published, study design, and level of evidence; sport played; and outcome measures from the studies themselves. Methodological quality of the articles was assessed using the Methodological Index for Non-Randomized Studies (MINORS) criteria by two independent reviewers (J.W.K. and S.A.).^10^ MINORS scoring was applied to all articles with patients. Data were aggregated for qualitative and descriptive analyses. The authors also present their recommended return-to-sport algorithm.

## Results

Twenty-three articles met the criteria for inclusion (Fig. 1).^3^^,^^5^^,^^11^, ^12^, ^13^, ^14^, ^15^, ^16^, ^17^, ^18^, ^19^, ^20^, ^21^, ^22^, ^23^, ^24^, ^25^, ^26^, ^27^, ^28^, ^29^, ^30^ Eleven (47.8%) articles were patient case series (level IV evidence), and 12 (52.2%) articles were expert opinions and recommendations on RTP (level V evidence).^3^^,^^5^^,^^11^, ^12^, ^13^, ^14^, ^15^, ^16^, ^17^, ^18^, ^19^, ^20^, ^21^, ^22^, ^23^, ^24^, ^25^, ^26^, ^27^, ^28^, ^29^, ^30^, ^31^

Of the case series studies, six (54.5%) were published in 2014 or after.^22^^,^^23^^,^^27^, ^28^, ^29^, ^30^ The MINORS score for the patient-based studies, each of which was noncomparative, averaged at 9.4 (range, 7–11) (Table 1).

**Table 1:** Summary of RTP and Performance Data

Author, Year∗	No. of Patients	Follow-Up Time	MINORS Score	Sport	Surgical Technique	RTP Time frame	Performance Data	Complications
McCue et al,^11^ 1974	41	Not stated	8	Mix of sports (football, wrestling, skiing, baseball, basketball, lacrosse, polo, softball, and horse jumping)	“Reattachment of the ligament to periosteum and bone with a pull-out wire”	NA	40/41 (97.6%) RTP at preinjury level	1/40 (2.5%) with osteoarthritis, weakness, stiffness, and pain of the MCP joint
Derkash et al,^12^ 1987	69	31.6 mo (range, 16–46 mo)	7	Skiing	Suture button + prolene 3-0 prolene	NA	66/69 (96%) RTP at preinjury level Mild weakness of pinch in 31/69 (44.9%), moderate weakness in 2/69 (2.9%), and severe weakness in 1/69 (1.4%)	3/69 (4.4%) could not RTP because of pain or fear of reinjury
Lane,^13^ 1991	36	3.9 y (range, 2.0–8.5 y)	11	Mix of sports (football, tennis, skiing, and wrestling)	Old technique: pullout suture, K-wire fixation of joint New technique: suture to adductor pollicis or UCL remnant, K-wire fixation of bony avulsion, no fixation of joint	RTP *some* level, earlier in new-technique patients Old: 8.8 ± 2.3 wk New: 4.6 ± 1.4 wk RTP *previous* level, earlier in new-technique patients Old: 14.1 ± 4.0 wk New: 10.2 ± 2.6 wk	Old: 7/7 (100%) RTP at preinjury level. New: 29/29 (100%) RTP at preinjury level	Failure of repair at 2 wk in 1/36 (2.7%), rerupture in 1/36 (2.7%) at 9 mo
Zeman et al,^14^ 1998	45	18 mo (12–26 mo)	8	Skiing and mountain biking	Suture anchor + 2-0 PDS	Immediately after surgery	44/45 (97.8%) RTP at preinjury level and had no complaints of instability at MCP joint	7/45 (15.6%), numbness 3/45 (6.7%), pain with activities 12/45 (26.7%), reduced ROM
Badia,^24^ 2006	12	Mean, 34.2 mo (range, 12–84 mo)	7	Sport not specified	Arthroscopic debridement + K-wire fixation of the bony avulsion	8 wk permitted; all patients returned to activities within 3 mo	36/36 (100)% RTP at preinjury level	None
Werner et al,^22^ 2014	18	6 y (range, 2.5–9.5 y)	10	Football	Suture anchor + braided polyester suture	4 wk of nonskilled position, 7 wk of skilled position	18/18 (100%) RTP at preinjry level. No significant differences in *Quick*DASH scores for skill vs nonskill position players	None
Werner et al,^23^ 2017	26 (17 isolated UCL; 9 combined UCL/RCL)	Not stated	11	Football	Suture anchor, braided polyester suture	6 wk	26/26 (100%) RTP at preinjury level, including 17 isolated UCL and 9 combined UCL + RCL	None
Jack et al,^27^ 2018	21	At least 1 y	11	Baseball	NA	Total: 120.0 ± 75.9 d In season: 56.2 ± 15.0 d	21/21 (100%) RTP at preinjury level. Infielders had a lower rate of postoperative wins above replacement relative to before surgery. However, no differences in performance relative to controls, no difference based on hand dominance of injury, and no decrease in games per season or career length after injury	None
Sochacki et al,^28^ 2019	23	At least 1 y	10	Football	NA	Total: 132.2 ± 126.1 d In season: 34.8 d	22/23 (95.7%) RTP at preinjury level; 1-y NFL career survival rate of 87.0%. No differences in positions or compared with matched controls	1/23 (4.3%) failed medical physical examination after surgery, unknown if related to surgery
Gibbs and Shin,^30^ 2020	17	At least 1 y	10	Mix of sports (baseball, basketball, hockey, and volleyball)	Suture anchor, suture tape, braided polyester suture	Total: RTP some level, 50.5 ± 53.77 d RTP same level, 58.5 ± 56.31 In season: RTP some level, 30.9 ± 10.06 d RTP same level, 36.3 ± 11.22	17/17 (100%) RTP at preinjury level	None
Bernstein et al,^29^ 2020	3	1 y	10	Football	Suture anchor, braided polyester suture	13.3 ± 2.9 d	3/3 (100%) RTP at preinjury level	3 ipsilateral PIP joint dislocations in two-third patients (67%)

DASH, Disabilities of the Arm, Shoulder, and Hand; MINORS, Methodological Index for Non-Randomized Studies; NA, data unavailable; PDS, polydioxanone suture; PIP, proximal interphalangeal; RCL, radial collateral ligament.

∗ Articles are listed in chronological order.

### RTP and performance

The summary of RTP data in the 11 articles reporting on patients is provided in Table 1.^11^, ^12^, ^13^, ^14^^,^^22^, ^23^, ^24^^,^^27^, ^28^, ^29^, ^30^ The sports included were American football, soccer, basketball, baseball, skiing, hockey, and a mix of general/unspecified sports.

In total, 311 patients were included in patient-based articles. In general, the rate of RTP was high in all sports/articles, with all studies reporting a RTP rate of >96% and most reporting a RTP rate of 100%. In aggregate, the rate of RTP at the same level as preinjury after surgical treatment was 305 (98.1%) of 311. The RTP time frame ranged widely from immediately after surgery to >4 months in athletes out of season. Studies that evaluated athletes both in- and out-of-season reported sooner RTP in-season.^27^^,^^28^^,^^30^

In terms of performance, in addition to a high rate of return to a similar preoperative level of play, Jack et al^27^ and Sochacki et al^28^ found no performance metric detriments relative to matched controls in Major League Baseball (MLB) and National Football League (NFL) athletes, respectively.

The surgical technique that was used varied by patient series (Table 1). Wire fixation was used in three studies, each from 2006 or earlier.^11^^,^^13^^,^^24^ Braided sutures along with suture anchors were specifically noted in articles from 2014 and later.^22^^,^^23^^,^^29^^,^^30^

A total of 32 (10.3%) patients were reported to have postoperative complications. Only two (0.64%) patients, both in the study by Lane,^13^ were reported to have failure of repair or rerupture during the study period.

### Survey section and recommendations on RTP

Expert opinions and recommendations are summarized by sport in Table 2.^3^^,^^5^^,^^15^, ^16^, ^17^, ^18^, ^19^, ^20^, ^21^^,^^25^^,^^26^^,^^31^ The recommendations on timing to RTP vary by sport and author. Injuries with unstable bony components were recommended to be fixed with wires, screws, or tension band constructs by all authors commenting on these injuries.^3^^,^^15^^,^^16^^,^^18^^,^^21^ Some authors advocated for transfixing the MCP joint to ensure stability of the construct before beginning ROM exercises.^5^^,^^16^, ^17^, ^18^

**Table 2:** Summary of Expert Opinions/Recommendations on Return to Sport

Sport—Articles (Author, Year)∗	Surgical Technique	Rehabilitation	RTP Timing
Hockey			
Schroeder and Goldfarb,^3^ 2015	Suture anchor + nonabsorbable sutures. If bony fragment > 20% articular surface, fix with a K-wire or screw	Thumb spica cast for 4 wk after surgery, then hand-based thumb spica splint for 4 more wk	NA
Basketball			
Carlson,^17^ 2012	Suture anchor + nonabsorbable sutures. Transfix MCP joint with K-wire when early RTP is required	Hand-based thumb spica splint for 6 wk after surgery, then cut down the cone splint over the thumb for 6 wk	6–8 wk, shorter if can be splinted during play
De Giacomo and Shin,^31^ 2017	Suture anchors with suture tape augmentation	Plaster splint for 3 d. Hand-based thumb spica splint with beginning of ballhandling drills at 8 d after surgery. Strengthening, shooting, and position-specific drills begin at 3 mo	5 wk unprotected
Football			
Williams,^19^ 2012	Suture anchor	Hand-based casting or splinting for 2–3 wk with IPJ free, then begin ROM. Strengthening at 6 wk. Continue immobilization during play until 6–8 wk, and then athletic taping for the remainder of the season	2 wk to allow wound healing
Schroeder and Goldfarb,^3^ 2015	Suture anchor + nonabsorbable sutures. If bony fragment > 20% articular surface, fix with a K-wire or screw	Thumb spica cast for 4 wk, then hand-based thumb spica splint for 4 wk	NA
Baseball			
Chhor and Culp,^18^ 2012	Suture anchor + nonabsorbable sutures. K-wire to transfix MCP joint. A small screw or pin for avulsion fracture	Forearm-based thumb spica splint with IPJ free. Pin removal and ROM at 4 wk after surgery. Strengthening at 6–8 wk in the nonthrowing arm or 10–12 wk in the throwing arm	Nonpitcher RTP when ROM and strength 80% of contralateral. Pitcher RTP when ROM and strength 100% of contralateral
Schroeder and Goldfarb,^3^ 2015	Suture anchor + nonabsorbable sutures. If a bony fragment > 20% articular surface, fix with K-wire or screw	Nonthrowing arm: immobilization for 6 wk after surgery, then progressive ROM/strengthening with hand-based thumb spica splint or cutdown dorsal radial splint for 4 more wk Throwing arm: immobilization for 6 wk after surgery, then progressive ROM/strengthening	“In elite athletes, sport-specific algorithms may allow earlier return to play”
Sport unspecified			
Morgan and Slowman,^15^ 2001	No mention of the technique for ligamentous injury. For bony injury, recommend tension band wiring or interfragmentary screw	High-contact sport: Thumb spica gauntlet cast for 4 wk after surgery. Then, begin ROM and strengthening with a protective thermoplastic splint for 2 wk, followed by 6 more wk of rigid athletic taping Low-contact sport: Thumb spica gauntlet cast for 4 wk. Then, begin ROM and strengthening with a thermoplastic short opponens splint	Immediate
Johnson and Culp,^16^ 2009	Suture anchor + nonabsorbable sutures. Transfix MCP joint with a K-wire For fracture, fix with screw, pin, or tension band wiring	Thumb spica splint with IPJ free. At 7–10 d after surgery, remove sutures, begin IPJ ROM, and place in a thermoplastic thumb spica splint. At 4 wk, remove pin, begin MCP joint ROM, and continue splinting. At 6–8 wk, splint only during play. At 12 wk, discontinue splint during play, and continue athletic taping indefinitely	4 wk for protected play and 12 wk for unprotected play with taping
Ng and Hayton,^21^ 2013	Suture anchor For fracture, screw or tension band wiring	Full-time radial blocking splint for 6 wk with immediate flexion/extension with a therapist. Then, continue radial blocking splint only during play until 12 wk	Immediate
Dy et al,^20^ 2013	NA	NA	Protected play: 5/36 surgeons recommended RTP immediately after surgery, 20/36 surgeons recommended 2 wk, 10/36 surgeons recommended 6 wk, and 1/36 surgeons recommended 3 mo Surgeons treating football athletes tended to have earlier RTP, and those treating basketball athletes tended to have later RTP Unprotected play: 23/36 recommended 3 mo after surgery
Goldfarb et al,^25^ 2016	Suture anchor + nonabsorbable suture	Thumb spica cast for 3 wk after surgery, then early ROM. Thumb spica splint until 6–8 wk, and then cutout cone splint until 12 wk	2 wk to allow wound healing
Owings et al,^26^ 2016	Suture anchor. Midsubstance tears repaired directly with nonabsorbable braided sutures	Cast for 6 wk after surgery, then ROM and removable splint for 4 more wk. Strengthening begins at 8 wk after surgery	RTP “early on” if immobilization allowed and sport does not require use of the thumb
Avery et al,^5^ 2017	Suture anchor + nonabsorbable sutures. Transfix MCP joint with K-wires when early RTP is required	Hand-based thumb spica splint with IPJ free for 6 wk after surgery, and during sports until 3 mo. Finger and thumb IPJ motion begins immediately. MCP joint ROM begins at 6 wk	Immediately after surgery

NA, data not available; IPJ, interphalangeal joint.

∗ Articles are organized by sport. The article by Schroeder and Goldfarb^3^is listed multiple times throughout the table on the basis of recommendations by sport. The article by Dy et al^20^represents data from surveys of NFL, National Basketball Association, and Major League Baseball surgeons.

All authors recommended initial immobilization in rehabilitation and protection of the thumb when returning to sports.^3^^,^^5^^,^^15^, ^16^, ^17^, ^18^, ^19^, ^20^, ^21^^,^^25^^,^^26^^,^^31^ The timing of return varied. Some authors recommended specific time frames, such as after 2 weeks for wound healing or after several weeks.^16^^,^^17^^,^^19^^,^^25^^,^^31^ Immediate return to sporting activity was also suggested by several experts.^5^^,^^15^^,^^21^

On the other hand, RTP was also guided by sport, rehabilitation criteria, and hand dominance, particularly in baseball.^3^^,^^18^^,^^26^ Chhor and Culp^18^ stratified RTP criteria by position, requiring pitchers to have better strength and ROM relative to nonpitchers before RTP (100% strength and ROM relative to contralateral for pitchers vs 80% strength and ROM relative to contralateral for nonpitchers).

Dy et al^20^ surveyed team physicians in the NFL, the National Basketball Association (NBA), and MLB on RTP after thumb UCL tears. Although there was heterogeneity in the recommended time to RTP, most (20 of 36, 55.6%) recommended 2 weeks for protected RTP.^20^ Interestingly, those who treated football athletes were more likely to recommend earlier protected RTP than nonfootball-treating surgeons, and those who treated basketball athletes were less likely to recommend earlier protected RTP than nonbasketball surgeons.^20^ Accordingly, Williams^19^ recommended RTP at 2 weeks for football athletes and Carlson^17^ recommended RTP at 6 to 8 weeks for basketball athletes, although if they could play protected, play may be allowed sooner. For unprotected RTP, 23 of the 36 (63.9%) surveyed recommended waiting 3 months, which is similar to the protocol suggested by Johnson and Culp.^16^^,^^20^

## Discussion

The present systematic review summarizes data and recommendations in the literature on RTP for athletes sustaining injuries to the thumb UCL complex that were managed surgically. Overall, the included studies on patients were all level IV evidence and noncomparative in nature. Return-to-play rates were high, and athletes returned to pre-injury competitive levels regardless of the sport played. Few complications have been reported in the literature even in high-level and high upper extremity–demand athletes. Rehabilitation guidelines appear to vary by sport, technique, and author; some authors recommend time-based RTP, whereas others recommend metric-based RTP. This study, therefore, supports the authors’ hypotheses of high RTP rates, minimal postoperative performance detriments, and heterogeneity in RTP criteria.

The overall RTP rate in the literature exceeded 98% in aggregate. This rate of return is high relative to the RTP rates after orthopedic surgeries cited in the NBA and NFL.^32^, ^33^, ^34^ In NBA players, one of the most reliable RTP rates previously investigated is that after hand or wrist fractures, noted to be 98.1%.^32^^,^^33^ Similarly, a study in NFL athletes found one of the highest RTP rates after orthopedic surgery to be 96.3% after forearm fracture open reduction internal fixation.^34^ In general, it appears that although hand and wrist injuries may ostensibly be intricately related to sport, players fare well after appropriate treatment.

For those who did return to sports, all athletes returned at the same level as that before injury. Additional performance metrics were scarce in the available literature relative to other procedures. For example, many other data on performance after injury or surgery delve into game-play statistics and career longevity.^32^, ^33^, ^34^, ^35^, ^36^ In the NFL, thumb UCL sprains may account for 4% of hand and digital injuries and have been reported to result in a mean of 23 days of missed play.^37^ Sochacki et al^28^ found that after thumb UCL surgery in NFL athletes, they had no decrease in games per season or career length, and data did not differ by position or relative to matched controls. In MLB, Jack et al^27^ found infielders to have a lower rate of postoperative wins above replacement relative to that before surgery; however, these authors did not find a decrease in games per season, career length, or other performance statistics relative to matched controls, and there was no difference based on hand dominance of the injury.^27^ Future studies on thumb UCL injuries treated surgically in athletes should continue to evaluate sport-specific data, which may guide trainers and coaches to target areas prone to performance detriment and may direct counseling of athletes on postoperative expectations. In addition, specific combined injuries, such as combined UCL/radial collateral ligament injuries, as seen in NFL athletes or with MCP joint dislocations, require further study and comparison to isolated UCL injuries.^23^^,^^38^

Although not clearly apparent from the included studies, recent data are encouraging for early motion after surgical treatment of thumb UCL injuries. Biomechanical data suggest the safety of controlled active motion therapy after surgical repair of the thumb UCL.^39^ Furthermore, several studies support better outcomes with early mobilization than with immobilization. Those with earlier motion protocols may have quicker return to work, similar or better ultimate ROM, better pinch strength, and fewer complications.^40^^,^^41^ In concordance with the trend to earlier motion, fewer of the recent studies included in this review incorporated MCP joint immobilization with temporary Kirschner wires, although some authors continue to advocate for its use to provide stabilization for earlier RTP.^5^^,^^16^, ^17^, ^18^ Earlier motion protocols are also facilitated by the use of suture anchors, which were more commonly used in the recent studies.

In addition to suture anchors with braided sutures, suture tape augmentation is gaining in popularity for thumb UCL repair.^8^^,^^30^^,^^31^^,^^42^, ^43^, ^44^, ^45^, ^46^ Suture tape augmentation appears to provide superior biomechanical strength in terms of stiffness and load-to-failure than suture anchors or graft reconstructions.^42^^,^^46^ The advantage of additional strength immediately after surgery is the provision of inherent stability before the effects of biologic healing.^46^ On the other hand, concerns have been raised regarding mechanical stress shielding and its effect on the ultimate strength of the ligament.^47^ Thus far, studies on suture tape augmentation for thumb UCL injuries have all been favorable in terms of permitting early motion and, therefore, facilitating return to sports, and these findings appear similar to the early biomechanical and clinical results on the use of tape augmentation for injuries such as elbow UCL and ankle instability.^8^^,^^30^^,^^31^^,^^42^, ^43^, ^44^, ^45^, ^46^^,^^48^, ^49^, ^50^, ^51^, ^52^ However, the current study included only two articles that employed suture tape augmentation because there remains a dearth of literature on the long-term outcomes for the suture tape technique. Therefore, more longitudinal studies are needed to determine whether suture tape augmentation will become the standard of operative care for high-level athletes. It is possible that thumb spica casting and bracing may increase the risk of nearby joint dislocations; hence, earlier motion may also prevent additional injury.^29^

**Figure 2:**
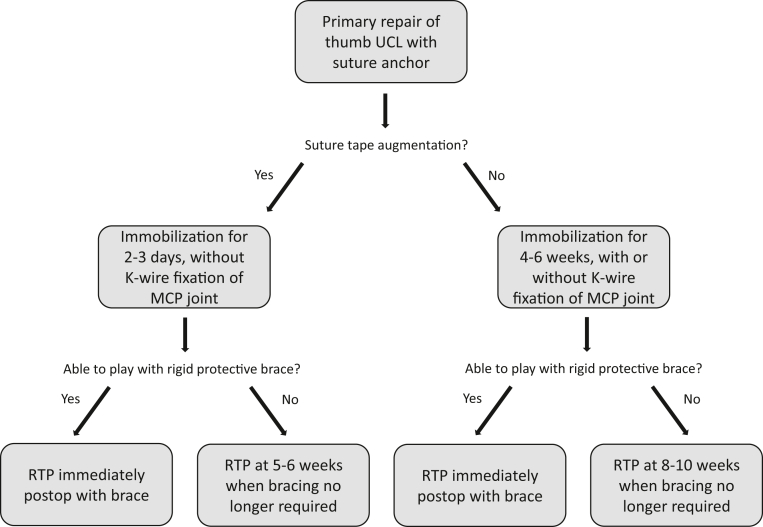
The authors’ recommended postoperative treatment and return-to-play (RTP) algorithm after thumb ulnar collateral ligament (UCL) repair in athletes. The recommendations in this flowchart represent our experience in consideration of the data reviewed in this study. MCP, metacarpophalangeal.

Based on this review of extant data and recent trends in surgical technique in combination with our own experience with the treatment of thumb UCL injuries, we believe that primary repairs using suture anchors can be treated appropriately with 4–6 weeks of immobilization, with or without additional stabilization through MCP joint pinning, followed by hand therapy. If suture tape augmentation is additionally used as an internal brace, the authors typically recommend postoperative immobilization of the thumb for 2–3 days after surgery without the need for MCP joint transfixion and also followed by a course of hand therapy. In our experience, we allow athletes to RTP as soon as they are able if they can play with a cast or rigid protective brace. Otherwise, they can RTP when bracing is no longer required. A summary of the authors’ treatment algorithm is demonstrated in Figure 2.

### Limitations

There are multiple limitations to this review, many of which are inherent to the included articles. Although the data compiled here support high RTP rates, minimal performance detriments, and low complication rates, they are limited by a low level of evidence. All studies that met the inclusion criteria were retrospective case series or expert opinions. The low level of evidence of data on injuries to the thumb UCL has been previously noted.^53^^,^^54^ Accordingly, we found the MINORS scores of the included studies to be poor. Future prospective studies comparing techniques would be beneficial to discerning the optimal ways to return to sport.

Additionally, because the data were heterogeneous among studies, aggregation of quantitative outcomes was limited. For example, the surgical techniques, sports, treatments, and rehabilitation protocols all differed among articles. Moreover, the included studies were level IV or V evidence and noncomparative, limiting the ability to draw comparative conclusions. This review-aggregated data and recommendations only related to athletes and did not capture information for those seeking to return to work and not return to sport. Furthermore, we incorporated only studies that discussed surgical management of thumb UCL injuries; nonsurgical treatment still plays an important role in treatment and RTP, especially for lower-grade injuries or partial tears of the UCL. Finally, although many of the injuries included were described as acute, the chronicity of injuries treated varied by study.

Return-to-play rates after surgical treatment of thumb UCL injuries are high, with reassuring return to the preinjury level of play with few complications. Recommendations for surgical technique have trended toward suture anchors and, now, suture tape augmentation with earlier motion protocols, although rehabilitation guidelines vary by sport and author. Current information on thumb UCL surgery in athletes is limited by the low quality of evidence and expert recommendations.
